# Clinical implications of expression of vascular endothelial growth factor in metastatic lesions of ovarian cancers

**DOI:** 10.1054/bjoc.2001.1933

**Published:** 2001-08

**Authors:** J Fujimoto, H Sakaguchi, I Aoki, S Khatun, T Tamaya

**Affiliations:** Department of Obstetrics and Gynecology, Gifu University School of Medicine, 40 Tsukasa-machi, Gifu City, 500–8705, Japan

**Keywords:** VEGF, metastatic lesion, patient prognosis, ovarian cancer

## Abstract

Vascular endothelial growth factor (VEGF) has been identified as an important factor for tumour angiogenesis, which is essential for the growth, invasion and metastasis of solid tumours. Significantly increased VEGF level from the primary tumour to the metastatic lesion of ovarian cancers was found in 8 of 30 cases. The 24-month survival rate of the patients with significantly increased VEGF level was extremely poor (0/8 = 0%) in comparison with that of patients with no change in the level (15/22 = 68%) from the primary tumour to the metastatic lesion. This indicates that VEGF may contribute to the advancement of metastatic lesions, and that VEGF level in metastatic lesions may be a prognostic indicator. © 2001 Cancer Research Campaign http://www.bjcancer.com


*British Journal of Cancer* (2001) **85**(3), 313–316
© 2001 Cancer Research Campaign
doi: 10.1054/ bjoc.2001.1933, available online at http://www.idealibrary.com on IDEAL®

### Clinical implications of expression of vascular endothelial growth factor in metastatic lesions of ovarian cancers

**J Fujimoto, H Sakaguchi, I Aoki, S Khatun and T Tamaya**

Department of Obstetrics and Gynecology, Gifu University School of Medicine, 40 Tsukasa-machi, Gifu City 500–8705, Japan

**Summary** Vascular endothelial growth factor (VEGF) has been identified as an important factor for tumour angiogenesis, which is essential for the growth, invasion and metastasis of solid tumours. Significantly increased VEGF level from the primary tumour to the metastatic lesion of ovarian cancers was found in 8 of 30 cases. The 24-month survival rate of the patients with significantly increased VEGF level was extremely poor (0/8 = 0%) in comparison with that of patients with no change in the level (15/22 = 68%) from the primary tumour to the metastatic lesion. This indicates that VEGF may contribute to the advancement of metastatic lesions, and that VEGF level in metastatic lesions may be a prognostic indicator. © 2001 Cancer Research Campaign http://www.bjcancer.com

**Keywords**: VEGF; metastatic lesion; patient prognosis; ovarian cancer

Neovascularization is essential for growth of and nutrition to solid tumours greater than 2 mm in diameter (Folkman, 1985). Among angiogenic factors, VEGF was initially recognized as a vascular permeability factor (34–42 kDa) that induced tumour ascites (Senger et al, 1983). Afterward, VEGF was identified as a vascular permeability factor that is active in increasing blood vessel permeability, endothelial cell growth and angiogenesis (Leung et al, 1989). Similarly, it was described as having a direct-acting mitogen specific for vascular endothelial cells (Keck et al, 1989). VEGF is expressed in tissues with rapid vascular endothelial turnover, which are reproductive organs such as ovary, uterus and placenta, and in tumours (Garrido et al, 1993; Li et al, 1994; Jackson et al, 1994). The level of VEGF correlates with microvessel density, but not with worsened patient prognosis in uterine cervical cancers (Fujimoto et al, 1999a). The expression of VEGF was down-regulated with advancement of uterine endometrial cancers lacking sex steroidal dependency (Fujimoto et al, 1998a, 1999b). In ovarian cancers, the expression of VEGF has been well demonstrated (Boocock et al, 1995; Abu-Jawdeh et al, 1996; Paley et al, 1997), and since the elevation of VEGF in ovarian cancers correlates with worsened patient prognosis, VEGF in the primary tumour of ovarian cancers is recognized as a prognostic indicator (Fujimoto et al, 1998b). On the other hand, although the presence of peritoneal metastasis in ovarian cancers is critical to patient prognosis (Eisenkop et al, 1993; Kapp et al, 1999), there is as yet no prognostic indicator for peritoneal metastasis-positive patients. This status prompted us to investigate the clinical significance of VEGF expression in peritoneal metastatic tumours of ovarian cancers.

*Received 11 August 2000*
*Revised 14 April 2001*
*Accepted 2 May 2001*

*Correspondence to:* J Fujimoto

### MATERIALS AND METHODS

### Patients

Informed consent for the following studies were obtained from all patients and the Research Committee for Human Subjects, Gifu University School of Medicine. Thirty patients ranging from 32 to 74 years of age underwent operation for ovarian cancer stage III at the Department of Obstetries and Gynecology, Gifu University School of Medicine between January 1995 and January 1998 (Table 1). None of the patients had received any preoperative therapy. A part of the tissues of ovarian cancers (the peritoneal metastatic lesion and the corresponding primary tumour) was obtained immediately after the resection and snap-frozen in liquid nitrogen to determine the levels of VEGF, and a neighbouring part of the tissues was submitted for histopathological study. The histological types and clinical stages were determined by International Federation of Obstetrics and Gynecology (FIGO) classification (FIGO News, 1989). Twenty-four-month survival rates were calculated for the 30 patients, all of whom underwent curative resection for ovarian cancer that achieved macroscopically disease-free status, and analyzed using the Kaplan–Meier method.

### Immunohistochemistry

Four-μm sections were cut from formalin-fixed paraffin-embedded tissue with a microtome and dried overnight at 37˚C on a silanized-slide (Dako, Carpinteria, CA, USA). Samples were deparaffinized in xylene at room temperature for 80 min and washed with a graded ethanol/water mixture and then with distilled water. The samples for VEGF were soaked in a citrate buffer and then microwaved at 100˚C for 10 min. The protocol for DAKO LSAB2 Kit, Peroxidase (Dako) was followed for each sample. In the described procedure, rabbit anti-human VEGF antigen VEGF(147) (200 μg/ml, Santa Cruz Biotechnology, Santa Cruz, CA, USA) as the first antibody was used at a dilution of

**Table 1** Patient details

| Case | Age (years) | Stage | Histology | Presence of tumour | VEGF |
|---|---|---|---|---|---|
| 1 | 39 | III | Serous cyAd, G1 | Ov, Pd (Il, Aw), Om, Ly | Increased |
| 2 | 71 | III | Serous ppcyAd, G3 | Ov, Pd (Il, Sc), Ly | Increased |
| 3 | 55 | III | Mucinous cyAd, G1 | Ov, Pd (Il, Sc), Om, Ly | Increased |
| 4 | 71 | III | Endometrioid Ad, G1 | Ov, Pd (Il, Cc, Sc, Lv, Aw), Om, Ly | Increased |
| 5 | 62 | III | Serous cyAd, G1 | Ov, Pd (Il, Sc, Aw), Ly | Increased |
| 6 | 35 | III | Mucinous cyAd, G1 | Ov, Pd (Il, Sc, Lv, Aw), Om, Ly | Increased |
| 7 | 63 | III | Serous ppcyAd, G2 | Ov, Pd (Sc), Il, Om, Ly | Increased |
| 8 | 69 | III | Clear cell Ad | Ov, Pd (Il, Cc, Sc), Ly | Increased |
| 9 | 54 | III | Clear cell Ad | Ov, Pd (Cc, Sc), Il, Ly | NC |
| 10 | 63 | III | Endometrioid Ad, G1 | Ov, Pd (Il, Sc, Aw), Om, Ly | NC |
| 11 | 50 | III | Serous ppcyAd, G1 | Ov, Pd (Il, Sc, Aw), Om, Ms, Ly | NC |
| 12 | 67 | III | Mucinous cyAd, G2 | Ov, Pd (Il, Aw), Om, Ly | NC |
| 13 | 56 | III | Mucinous cyAd, G1 | Ov, Pd (Sc, Aw), Il, Om, Ly | NC |
| 14 | 34 | III | Serous cyAd, G3 | Ov, Pd (Jj, Sc, Aw), Il, Om, Ms, Ly | NC |
| 15 | 49 | III | Endometrioid Ad, G1 | Ov, Pd (Il, Sc, Aw), Om, Ly | NC |
| 16 | 50 | III | Endometrioid Ad, G1 | Ov, Pd (Il, Cc, Aw), Om, Ly | NC |
| 17 | 54 | III | Mucinous cyAd, G1 | Ov, Pd (Il, Sc, Aw), Ly | NC |
| 18 | 48 | III | Endometrioid Ad, G1 | Ov, Pd (Sc, Aw), Om, Ly | NC |
| 19 | 59 | III | Clear cell Ad | Ov, Pd (Sc, Aw), Il, Om, Ly | NC |
| 20 | 68 | III | Serous cyAd, G1 | Ov, Pd (Cc, Sc), Ly | NC |
| 21 | 60 | III | Serous pcyAd, G1 | Ov, Pd (Il, Sc, Lv, Aw), Om, Ly | NC |
| 22 | 74 | III | Serous cyAd, G1 | Ov, Pd (Il, Cc), Ly | NC |
| 23 | 49 | III | Serous ppcyAd, G1 | Ov, Pd (Il, Sc, Aw), Om, Ms, Ly | NC |
| 24 | 71 | III | Clear cell Ad | Ov, Pd (Il, Cc, Sc), Om, Ly | NC |
| 25 | 50 | III | Mucinous cyAd, G1 | Ov, Pd (Il, Aw), Ly | NC |
| 26 | 64 | III | Serous cyAd, G1 | Ov, Pd (Il, Sc, Aw), Om, Ly | NC |
| 27 | 67 | III | Mucinous cyAd, G2 | Ov, Pd (Sc, Aw), Il, Om, Ly | NC |
| 28 | 69 | III | Endometrioid Ad, G2 | Ov, Pd (Il, Cc, Aw), Om | NC |
| 29 | 44 | III | Mucinous cyAd, G1 | Ov, Pd (Sc) | NC |
| 30 | 67 | III | Serous cyAd, G1 | Ov, Pd (Il, Sc, Aw), Ly | NC |

Ov, ovaries; Pd, peritoneal dissemination; Il, ileum; Cc, caecum; Jj jejunum; Lv, liver; Aw, abdominal wall; Sc, sigmoid colon; Om, omentum; Ms, mesentery; Ly, pelvic lymph nodes G1, well-differentiated; G2, moderately differentiated, G3, poorly differentiated; Ad, adenocarcinoma; cyAd, cystadenocarcinoma; ppcyAd, papillary cystadenocarcinoma; Increased, cases with significantly increased VEGF level from the primary tumour to the peritoneal metastatic lesion; NC, cases with no change in VEGF level from the primary tumour to the peritoneal metastatic lesion.

1:100. The antibody against a peptide corresponding to amino acids 1–147 of VEGF can detect all isoforms of VEGF (wild type VEGF $_{206}$, VEGF $_{189}$, VEGF $_{165}$, and VEGF $_{121}$). The addition of the first antibody was omitted in the protocol for negative controls.

### Enzyme immunoassay for determination of human VEGF antigen

All steps were carried out at 4°C. Tissues (wet weight: 10–20 mg) were homogenized in HG buffer (5 mM Tris-HCl, pH 7.4, 5 mM NaCl, 1 mM $CaCl_2$, 2 mM ethyleneglycol-bis-[β-aminoethyl ether]-N,N,N′, N′-tetraacetic acid, 1 mM $MgCl_2$, 2 mM dithiothreitol, 25 μg/ml aprotinin, and 25 μg/ml leupeptin) with a Polytron homogenizer (Kinematics, Luzern, Switzerland). This suspension was centrifuged in a microfuge at 12 000 rpm for 3 min to remove the nuclear pellet. The protein concentration of samples was measured by the method of Bradford to standardize VEGF antigen levels (Bradford, 1976).

VEGF antigen levels in the samples were determined by a sandwich enzyme immunoassay using a Human VEGF Assay Kit-IBL (Immuno Biological Laboratories, Gunma, Japan). The levels of VEGF were standardized with the corresponding cellular protein concentrations.

### Statistics

The levels of VEGF were measured from three parts of the same tissue of each primary tumour and metastatic lesion in triplicate (9 determinations each; 18 determinations for each case). The *t*-test for two independent samples was used in a one-tail manner to compare the nine determinations for each primary tumour against the nine determinations for each metastatic lesion (30 independent *t*-tests). Survival curves were calculated using the Kaplan–Meier method, and analyzed by the log-rank test. Differences were considered significant when $P$ was less than 0.05.

### RESULTS

Immunohistochemical staining for VEGF ($n = 30$) was carried out to study VEGF localization and the intensity of staining in the peritoneal metastatic lesion and the corresponding primary tumour. As shown in Figure 1, positive staining is seen dominantly in the cytoplasm of the cancer cells, and faintly in interstitial cells. There was no case of decreased intensity from the primary tumour to the metastatic lesion. Obviously increased intensity from the primary tumour to the metastatic lesion was found in 8 of 30 cases of ovarian cancers.

Enzyme immunoassay for VEGF was carried out to study VEGF level in the peritoneal metastatic lesion and the corresponding primary tumour. As shown in Figure 2, there was no case of decreased VEGF level from the primary tumour to the metastatic lesion. Significantly, ($P < 0.05$) increased VEGF level from the primary tumour to the metastatic lesion was found in 8 of 30 cases, the same cases that showed increased intensity of immunohistochemical staining for VEGF.

 *© 2001 Cancer Research Campaign*

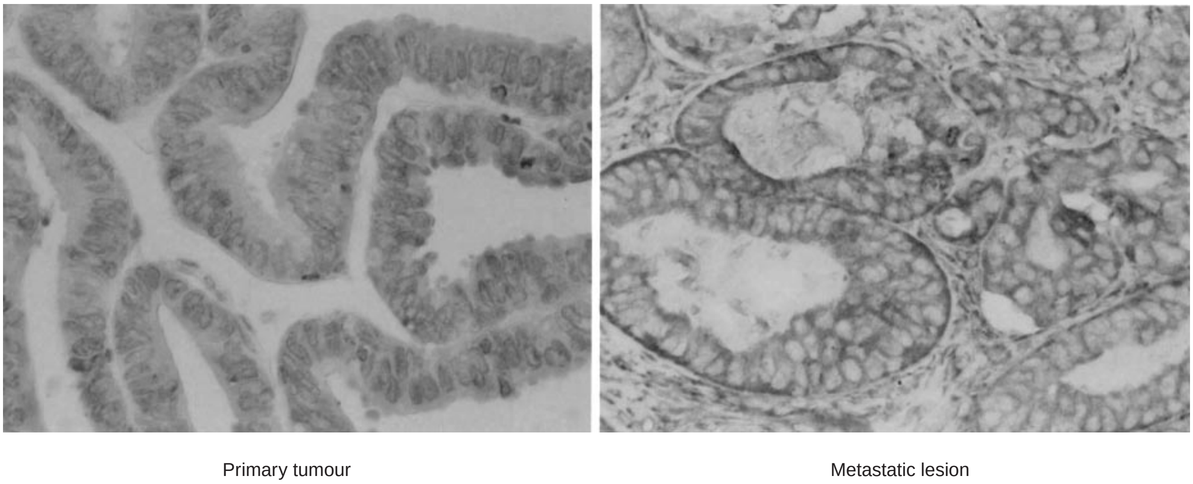

**Figure 1** Immunohistochemical staining for VEGF in peritoneal metastatic lesion and corresponding primary tumour of ovarian cancers. This is a representative case (case 5, serous cystadenocarcinama shown in Figure 2) with increased intensity from the primary tumour to the metastatic lesion

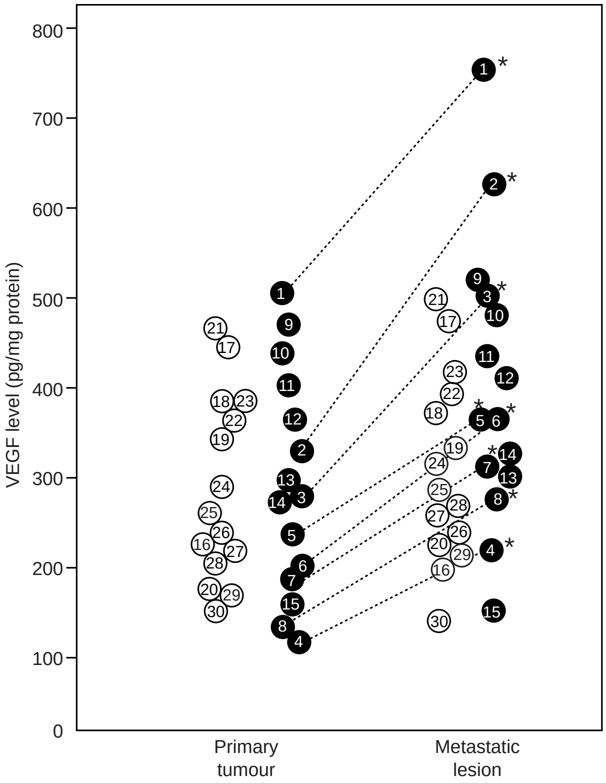

**Figure 2** Levels of VEGF in peritoneal metastatic lesion and corresponding primary tumor of ovarian cancers. The levels of VEGF were determined by a sandwich enzyme immunoassay using a Human VEGF Assay Kit-IBL (Immuno Biological Laboratories, Gunma, Japan). Each result is the mean of 9 determinations. Number in ○, living case; number in ●, deceased case. *$P < 0.05$ vs the corresponding primary tumour

We analysed the prognosis of the 30 patients who underwent curative resection and whose 24-month survival rates were calculated. As shown in Figure 3, the prognosis of patients with significantly increased VEGF level was significantly ($P < 0.05$) poor (0.8 = 0%) compared to that of patients with no change in the level (15/22 = 68%) from the primary tumour to the peritoneal metastatic lesion.

### DISCUSSION

Newly developed capillary network formation from the original vessel is designated as neovascularization. Generally, turnover of capillary endothelial cells is extremely slow to the order of months or years in physiological neovascularization, while the turnover in ovary and uterine endometrium is altered to a rapid state within the ovarian cycle. The turnover with malignant transformation becomes rapid, which might contribute to the acceleration of tumour growth (Denekamp, 1984).

Among angiogenic factors, VEGF has been evaluated as an important factor for tumour angiogenesis, which is essential for the growth of solid tumours. Generally speaking, VEGF secreted from tumours contributes to tumour growth not via an autocrine pathway to tumour cells, but via a paracrine pathway to surrounding microvessels (Berkman et al, 1993). The elevation of VEGF in ovarian cancers correlates with worsened patient prognosis (Fujimoto et al, 1998b). On the other hand, KDR, a receptor

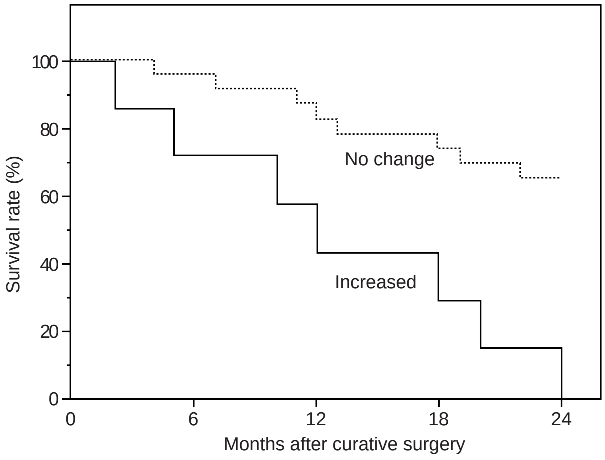

**Figure 3** Survival rates after curative resection for ovarian cancer. Patient prognosis was analysed with a 24-month survival rate. Increased: the cases with significantly increased VEGF level from the primary tumour to the peritoneal metastatic lesion. No change; the cases with no change in VEGF level from the primary tumour to the peritoneal metastatic lesion

© 2001 Cancer Research Campaign of VEGF, is expressed by some ovarian cancer cells that co-express VEGF (Boocock et al, 1995). Co-expression of VEGF and KDR by tumour cells in ovarian cancer raises the possibility of autocrine stimulation (Boocock et al, 1995). Furthermore, it facilitates metastasis via neovascularization (Warren et al, 1995). Although the presence of peritoneal abdominal metastasis is critical to patient prognosis, there is as yet no prognostic indicator for peritoneal metastasis-positive patients in ovarian cancers.

In the present study, it was demonstrated that the prognosis of patients with increased VEGF level from the primary tumour to the peritoneal metastatic lesion was extremely poor in comparison with that of patients with no change in the level. Thus, VEGF may contribute to the advancement of metastatic lesions, and VEGF level in metastatic lesions may be a prognostic indicator. This indicates that anti-VEGF and VEGF receptor antibodies (Borgstrom et al, 1996; Yuan et al, 1996; Zhu et al, 1998; Prewett et al, 1999) might be effective against advanced ovarian cancers, especially the cases with increased VEGF level from the primary tumour to the metastatic lesion, apart from a direct anti-tumoural effect on cancer cells.

### REFERENCES

Abu-Jawdeh GM, Faix JD, Niloff J, Tognazzi K, Manseau E, Dvorak HF and Brown LF (1996) Strong expression of vascular permeability factor (vascular endothelial growth factor) and its receptor in ovarian borderline and malignant neoplasms. *Lab Invest* **74**: 1105–1115

Berkman RA, Merrill MJ, Reinhold WC, Monacci WT, Saxena A, Clark WC, Robertson JT, Ali IU and Oldfield EH (1993) Expression of vascular permeability factor/vascular endothelial growth factor gene in central nervous system neoplasms. *J Clin Invest* **91**: 153–159

Boocock CA, Charnock-Jones DS, Sharkey AM, McLaren J, Barker PJ, Wright KA, Twentyman PR and Smith SK (1995) Expression of vascular endothelial growth factor and its receptor flt and KDR in ovarian carcinoma. *JNCI* **87**: 506–516

Borgstrom P, Hillan KJ, Sriramarao P and Ferrara N (1996) Complete inhibition of angiogenesis and growth of microtumors by anti-vascular endothelial growth factor neutralizing antibody: novel concepts of angiogenic therapy from intravital videomicroscopy. *Cancer Res* **56**: 4032–4039

Bradford M (1976) A rapid and sensitive method for the quantitation of microgram quantities of protein utilizing the principle of protein-dye binding. *Anal Biochem* **72**: 315–323

Denekamp J (1984) Vascular as a target for tumor therapy. *Prog Appl Microcirc* **4**: 28–38

Eisenkop SM, Nalick RH, Wang HJ and Teng NN (1993) Peritoneal implant elimination during cytoreductive surgery for ovarian cancer: impact on survival. *Gynecol Oncol* **51**: 224–229

FIGO News (1989) *Int J Gynecol Obstet* **28**: 189–193

Folkman J (1985) Tumor angiogenesis. *Adv Cancer Res* **43**: 175–203

Fujimoto J, Ichigo S, Hirose R, Sakaguchi H and Tamaya T (1998) Expression of vascular endothelial growth factor (VEGF) and its mRNA in uterine endometrial cancers. *Cancer Lett* **134**: 15–22

Fujimoto J, Sakaguchi H, Hirose R, Ichigo S and Tamaya T (1998) Biological implication of the expression of vascular endothelial growth factor (VEGF) subtypes in ovarian cancers. *Cancer* **83**: 2527–2533

Fujimoto J, Sakaguchi H, Hirose R, Ichigo S and Tamaya, T (1999a) Expression of vascular endothelial growth factor (VEGF) and its mRNA in uterine cervical cancers. *Br J Cancer* **80**: 827–833

Fujimoto J, Sakaguchi H, Hirose R, Ichigo S and Tamaya T (1999b) Progestins suppress estrogen-induced expression of vascular endothelial growth factor (VEGF) subtypes in uterine endometrial cancer cells. *Cancer Lett* **141**: 63–71

Garrido C, Saule S and Gospodarowicz D (1993) Transcriptional regulation of vascular endothelial growth factor gene expression in ovarian bovine granulosa cells. *Growth Factors* **8**: 109–117

Jackson MR, Carney EW, Lye SJ and Knoxritchie JW (1994) Localization of two angiogenic growth factors (PDECGF and VEGF) in human placentae throughout gestation. *Placenta* **15**: 341–353

Kapp KS, Kapp DS, Poschauko J, Stucklschweiger GF, Hackl A, Pickel H, Petru E and Winter R (1999) The prognostic significance of peritoneal seeding and size of postsurgical residual in patients with stage III epithelial ovarian cancer treated with surgery, chemotherapy, and high-dose radiotherapy. *Gynecol Oncol* **74**: 400–407

Keck PJ, Hauser SD, Krivi G, Sanzo K, Warren T, Feder J and Connolly DT (1989) Vascular permeability factor, an endothelial cell mitogen related to PDGF. *Science* **246**: 1309–1312

Leung DW, Cachianes G, Kuang WJ, Goeddel DV and Ferrara N (1989) Vascular endothelial growth factor is a secreted angiogenic mitogen. *Science* **246**: 1306–1309

Li XF, Gregory J and Ahmed A (1994) Immunolocalization of vascular endothelial growth factor in human endometrium. *Growth Factors* **11**: 277–282

Paley PJ, Staskus KA, Gebhard K, Mohanraj D, Twiggs LB, Carson LF and Ramakrishnan S (1997) Vascular endothelial growth factor expression in early stage ovarian carcinoma. *Cancer* **80**: 98–106

Prewett M, Huber J, Li Y, Santiago A, O'Connor W, King K, Overholser J, Hooper A, Pytowski B, Witte L, Bohlen P and Hicklin DJ (1999) Antivascular endothelial growth factor receptor (fetal liver kinase 1) monoclonal antibody inhibits tumor angiogenesis and growth of several mouse and human tumors. *Cancer Res* **59**: 5209–5218

Senger DR, Galli SJ, Dvorak AM, Perruzzi CA, Harvey VS and Dvorak HS (1983) Tumor cells secrete a vascular permeability factor that promotes accumulation of ascites fluid. *Science* **219**: 983–985

Warren RS, Yuan H, Matli MR, Gillett NA and Ferrara N (1995) Regulation by vascular endothelial growth factor of human colon cancer tumorigenesis in a mouse of experimental liver metastasis. *J Clin Invest* **95**: 1789–1797

Yuan F, Chen Y, Dellian M, Safabakhsh N, Ferrara N and Jain RK (1996) Time-dependent vascular regression and permeability changes in established human tumor xenografts induced by an anti-vascular endothelial growth factor/vascular permeability factor antibody. *Proc Natl Acad Sci USA* **93**: 14765–14770

Zhu Z, Rockwell P, Lu D, Kotanides H, Pytowski B, Hicklin DJ, Bohlen P and Witte L (1998) Inhibition of vascular endothelial growth factor-induced receptor activation with anti-kinase insert domain-containing receptor single-chain antibodies from a phage display library. *Cancer Res* **58**: 3209–3214

*© 2001 Cancer Research Campaign*
